# Chicken eggs, childhood stunting and environmental hygiene: an ethnographic study from the *Campylobacter* genomics and environmental enteric dysfunction (CAGED) project in Ethiopia

**DOI:** 10.1186/s42522-020-00012-9

**Published:** 2020-03-23

**Authors:** Kevin Louis Bardosh, Jeylan Wolyie Hussein, Elias Ahmed Sadik, Jemal Yousuf Hassen, Mengistu Ketema, Abdulmuen Mohammed Ibrahim, Sarah Lindley McKune, Arie Hendrik Havelaar

**Affiliations:** 1 Department of Anthropology, University of Florida, Gainesville, USA; 2 Center for One Health Research, School of Public Health, University of Washington, Seattle, USA; 3 College of Social Sciences and Humanities, Haramaya University, Dire Dawa, Ethiopia; 4 Department of Rural Development and Agricultural Extension, Haramaya University, Dire Dawa, Ethiopia; 5 School of Agricultural Economics and Agribusiness Management, Haramaya University, Dire Dawa, Ethiopia; 6 Environmental and Global Health, University of Florida, Gainesville, USA; 7 Department of Animal Sciences, Emerging Pathogens Institute, Institute for Sustainable Food Systems, University of Florida, Gainesville, USA

**Keywords:** Poultry, Childhood stunting, Environmental hygiene, *Campylobacter*, One health, Ethiopia

## Abstract

**Background:**

Childhood stunting and malnutrition condemn millions of people globally to a life of disadvantage and cognitive and physical impairment. Though increasing egg consumption is often seen as an important solution for low and middle income countries (including Ethiopia), emerging evidence suggests that greater exposure to poultry feces may also inhibit child growth due to the effects of enteric bacteria, especially *Campylobacter*, on gut health.

**Methods:**

In this rapid ethnographic study, we explored village poultry production, child dietary practices, and environmental hygiene conditions as they relate to *Campylobacter* risk and intervention in 16 villages in Haramaya Woreda, Eastern Ethiopia.

**Results:**

In the study area, we found that women assumed primary responsibility to care for both chickens and children: in feeding, housing, and healthcare. Most chickens were free-range local indigenous breeds, and flock sizes were small and unstable due to epidemics, seasonal trends, reproductive patterns, and lack of food. Generally, eggs were seen as “too luxurious” to be eaten, and were predominantly sold at local markets for scarce cash, despite high malnutrition rates. Local narratives of extreme poverty, social dietary norms, parental fatalism, and lack of “dietary consciousness” (as it was called) were invoked to explain this. We found that homesteads were highly contaminated with human and animal feces. Although community members viewed chicken feces and poultry gastrointestinal contents as particularly noxious in comparison to other animals because of their feeding behaviour, they did not relate them to any particular disease. Shared human-animal housing and childcare practices place children at high risk of exposure to enteric bacteria from animal manure, despite daily routines designed to manage the domestic landscape.

**Conclusions:**

Addressing childhood stunting and malnutrition through egg production in rural landscapes like Haramaya must navigate three distinct health and care regimes: for children, chickens, and home environments. Interventions should be based on a holistic approach to social and economic empowerment, one that considers both women and men and integrates nutrition, health, and community change as its overarching goal.

## Introduction

Rates of extreme poverty and under-nutrition have dropped precipitately over the last 50 years [1]. However, two billion people around the world currently experience moderate to severe food insecurity and 149 million children are stunted [2]. Early life exposure to under-nutrition, including in the womb, has drastic influences on the health of individuals, communities and, indeed, whole nations [3, 4]. There are the immediate signs: mortality, delayed and permanently damaged neurological and physical growth, and greater susceptibility to infectious and chronic disease. But there are also more long-term, often hidden, whole-of-society effects: lost human capital, vanished economic productivity, and a general deterioration in human wellbeing.

Child under-nutrition remains a major problem in African countries such as Ethiopia [5]. The most recent Demographic and Health Survey (DHS) in 2016 found that 38% of children under five were suffering from chronic malnutrition (stunting or low height-for-age), a rate that rose to nearly 50% in some regions and is nearly equivalent to 6 million children [6]. As noted by Abdulahi et al. [7], a complex number of factors are involved: “child age, child sex, complementary food, poor dietary diversity, diarrheal diseases, maternal education, maternal height, residential area and socio- economic status.”

Ethiopia is one of the world’s largest countries, with 108 million people (in 2018), predicted to rise to 190 million by 2050. Although it remains a low-income country, with millions in extreme poverty, Ethiopia has achieved remarkable economic changes since 2000 [8]. The country is one of the fastest growing global economies, with an average 10% GDP growth from 2005 to 2016 [9].

Policy debates about hunger and childhood stunting have increasingly emphasized the importance of expanding production and access to animal-sourced foods, including chicken production [10]. In 2015, there were an estimated 60.5 million chickens in Ethiopia, 94% of which were local indigenous breeds kept in backyard smallholder systems (with an average flock size of less than five) and limited access to agricultural and veterinary inputs [11]. Ethiopia’s Livestock Master Plan (LMP), released in 2015, aimed to triple chicken production by 2020. This included an ambitious goal to increase egg production, from an estimated 419 million eggs per year (in 2014) to 3.8 billion by 2020 [12].

As Iannotti et al. [13] have argued, increasing egg production for countries like Ethiopia may be an “uncracked” solution to global childhood under-nutrition, providing essential fatty acids, proteins, vitamins, and other critical nutrients typically at levels above or equal to other animal-sourced food. However livestock intensification also comes with risks, notably the health consequences of zoonotic pathogens [14]. Animal excreta, particularly chicken feces, are major sources of enteric disease [15] and an important contributor to the death and under-development of children under-five due to diarrheal disease. In smallholder (“backyard”) poultry systems, human exposure to animal feces comes from many sources: fluids, fields, flies, fingers, fomites, and food [16]. A study in Zimbabwe found substantial ingestion of chicken feces-related pathogens in infants and young children due to poor hygiene behaviors and geophagy (eating dirt), specifically childhood mouthing and exploratory play [17]. Such epidemiological findings are representative of social and economic determinants of ill-health: poverty, malnutrition, gender inequality, poor housing, and a lack of clean water, hygiene, sanitation, and food safety.

In this paper, we investigate these issues through an exploratory ethnographic lens, as part of a multidisciplinary research project - *Campylobacter Genomics and Environmental Enteric Dysfunction (CAGED) in Ethiopia* – concerned with understanding the relationships between poor diets, zoonotic *Campylobacter spp*., chicken management, and childhood stunting in a rural district of Ethiopia.

*Campylobacter* is one of the most ubiquitous and significant enteric bacterial infections worldwide. More than 160 million people are infected each year, with 38,000 deaths around the world including a disproportionate burden in Africa [18]. The bacteria is widely endemic in Ethiopia; a recent study in Nuer Zone, Gambella, found *Campylobacter spp.* in fecal samples from 87% of chickens, 48% of cattle, 39% of sheep, and 33% of goats [19]. Importantly, only humans and other higher primates can develop clinical disease, and infection is usually mild and most are asymptomatic.

Emerging research, however, is now suggesting that *Campylobacter* colonization, through direct and indirect exposure to chicken droppings, has other health effects, notably as a cause of environmental enteric dysfunction (EED): the inflammation of the intestine, resulting in poor absorption of food, vitamins, and minerals. Contaminated living conditions where children and chicken feces intermix may, therefore, be an important contributing factor to global stunting rates.

A recent landmark study from eight low-resource countries revealed that a high *Campylobacter spp*. prevalence in primarily asymptomatic children was associated with a lower length-for-age z (LAZ) score, increased intestinal permeability, and intestinal and systemic inflammation at 24 months of age [20]. The realization that most stunting is not caused, exclusively at least, by poor diet, sanitation and/or diarrhea alone, together with increasing interest in “gut health”, is raising the profile of EED in global nutrition, development, and health circles [21].

*Campylobacter* resides in the gastrointestinal tract of poultry, ducks, turkeys, and other warm-blooded animals, where they are typically benign. Traditionally, the view has been that most human exposure occurs through transmission via food, making thoroughly cooking chicken meat and improving kitchen hygiene to prevent cross-contamination (from chicken meat to ready-to-eat foods) the dominant prevention strategies. Indeed, the WHO estimated that in high income countries, foodborne transmission accounts for 68–76% of all cases of human campylobacteriosis. In Africa, however, foodborne transmission is estimated to account for only 53% of all cases [22].

Rapidly rising demand for chicken meat and eggs may have the unintended consequence of increasing childhood exposure to greater quantities of *Campylobacter*. While attempting to reduce stunting rates through increased production of eggs or other animal- source foods, intensification policies may also simultaneously increase stunting rates, if animal excreta are not managed accordingly.

In this study, we used an informal ethnographic approach to investigate the local contexts and drivers of chicken keeping, child diet, and environmental hygiene in one district of southeastern Ethiopia. The study was part of a larger, formative research study that aims to lay the groundwork for a series of epidemiological and molecular investigations, as well as a possible randomized control trial (RCT) on the effects of an integrated poultry, hygiene, and nutrition intervention on *Campylobacter* and child stunting rates. Methodologically, our exploratory ethnographic work built on fieldwork conducted elsewhere, on other zoonotic diseases including echinococcosis in Morocco [23] and cysticercosis in Lao PDR [24]. Our aim was to understand the various practices that govern the care of chickens, children, and home environments and their interactions, and how these may influence poultry interventions and the spread of *Campylobacter*.

## Methods and study area

### Study methodology

Our formative study was conducted over 12-weeks in mid-2018 and based on a rapid ethnographic approach to data collection and analysis. The study was guided by two core objectives:

1. To understand local community contexts, socio-cultural beliefs and practices, and social organization in relation to poultry, dietary intake, WASH, and child growth as they pertain to *Campylobacter* epidemiology;
2. To explore community-level opportunities and barriers to possible interventions aimed at improving poultry biosecurity and zoonotic disease prevention, with a specific focus on caging poultry.

Data were collected through informal interviews, group discussions, participant and direct observations, and team debriefings, working across 16 villages in four kebeles (the smallest administrative unit in Ethiopia) in Haramaya District (Woreda), Eastern Ethiopia. Kebeles are further subdivided into informal villages with no separate administrative structure. In each kebele, four villages were included in the study, selected to maximize variations in social, demographic, and ecological characteristics. Each village was visited for three non-consecutive days at different times of the study. The study was designed and conducted by three experienced social scientists.

The study design included a total of 10 research topics, divided into three research themes: chicken management, WASH and nutrition, and social organization. The field team used these as the basis for interviews but also relied on more didactic methods: using field observations (and objects) as a starting point for conversations, asking hypothetical questions, seeking out stories and examples, triangulating particular events and information, and using imaginative and projective thinking. In all cases, interviews and group discussions were informal as the main goal was to explore what people said about these topics when they came up naturally and not in a formal interview setting.

The study also included a list of structured observations on the level of WASH at household level, malnutrition in children and a few direct demographic questions. Below, when we refer to these results, we will refer to our “household survey.” In each of the 16 villages, eight households with poultry were randomly selected for direct observation, for a total of *n* = 128 households. To determine signs of malnutrition in children, we relied on physical, observable signs of malnutrition such as hair discoloration, physical emaciation, head enlargement, old-looking or bruised faces, swelling on faces and abdomens of children (lower extremities as well) as well as household reports of children’s high susceptibility to chronic infection and ill-health. A senior medical nurse carried out this assessment.

An important methodological consideration within our ethnography was following a focused approach to data collection [25] particularly when we were interested in the health implication of human-animal interactions in general and children’s contact with chicken feces. Our ethnography focused on observing the inner workings of livelihood realities and collecting participants’ reflections on and insights into the meanings of human-animal interactions, the health implications of those interactions and their views on the economic value of poultry production and poultry-related diseases.

Data collection and analysis followed an iterative process. Each step of data collection involved rapid informal analysis on a daily basis that, in turn, provided useful insights on the importance of gathering further data and shaping ways of gathering it. Initial analysis and interpretation of data were discussed through peer debriefing, which enabled the study team to address methodological, procedural, conceptual, analytic, and interpretational issues whilst in the field. Data involved hand-written interview notes, observational notes, reflective notes and photographs. Formal analysis first involved a process of transcribing these notes into Microsoft Word and categorizing these data according to the three research themes (chicken management, WASH and nutrition, and social organization) and 10 research topics of the study. This was done for each of the four kebeles, producing four independent reports. These four reports were then merged, maintaining identification codes for location (kebele, village) and the socio-demographic details (age, sex, economic status) of respondents. Formal data analysis involved printing out the full repository of the data and coding the data by hand. An initial list of codes and sub-codes, for each research theme, was developed inductively from the data. Once a preliminary coding of the data was performed, it was shared between the ethnographic data collection team for review and validation. The data were then grouped according to kebele and respondent characteristics (age, sex, economic status) to explore any possible divergences and a final coding of the data was performed. All analytical categories were developed inductively from the data. Once the final coding and analysis was performed, the original reports were reviewed to ensure the reliability and fidelity of our analytical categories and interpretation. When we use quotation marks in this paper, we are highlighting terms that were widely used to describe local phenomena by community members.

### Study location

**Fig. 1:**
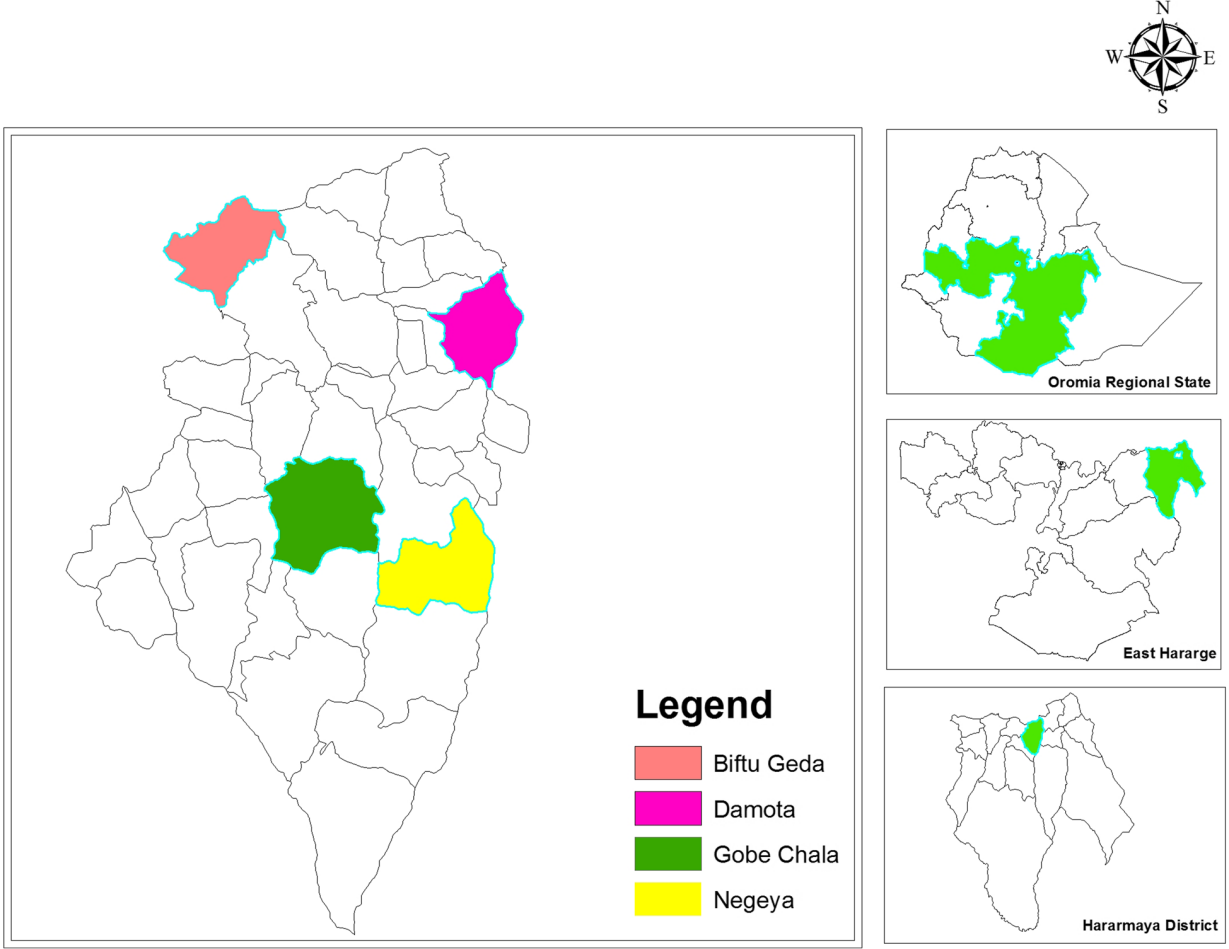
Map of the study area

Our study was conducted in four kebeles in Haramaya District, part of Oromia region. This included the kebeles of Damota, Arada Nagaya, Gobe Chala, and Biftu Geda (see Fig. 1).

Haramaya is a semi-arid ecological zone inhabited by sedentary Hararghe mixed crop-livestock farmers, almost all Muslim. Its a socio-ecological landscape under pressure from social, demographic, climate, and ecological changes that are threatening soil productivity and water and land availability. Our fieldwork clearly showed these pressures. Most valleys and hills are under cultivation, rainfall has become more erratic, there is less ground water, and farmers have also begun to cultivate lake basins. Family size and fertility rates are high, youth unemployment is widespread, and land fragmentation is leading to smaller and smaller plots of agricultural land, which makes it difficult to afford commodities from the increasingly urbanizing small towns. As a consequence, cattle populations are reducing while smaller ruminant populations (goats and sheep) are relatively increasing. The small ruminants are largely raised under zero-grazing conditions with limited supplementary feed.

Perhaps the most dramatic and significant change in Haramaya district over the last 10–15 years, confirmed during our fieldwork, has been the commercialization of khat (*Catha edulis*). Khat is a mildly stimulant plant of which leaves are chewed predominately by men to get a state of euphoria and excitement. A year-round, low-input export crop with high demand from the Horn of Africa and Arabian Peninsula, it has become the dominant cash-crop in Haramaya, moving many farmers away from cereal production. The fact that the entire landscape of the district is covered with khat year-round is suggestive of its growing economic and cultural importance. While khat is supplemented by small-scale vegetable production (especially potato, sweet potato, onions, tomatoes, and carrots), some of which is sold and some of which is consumed at household level, a good majority of people rely on selling their khat to purchase food from urban areas or local markets. Sometimes farmers are not able to recover their costs in agricultural inputs, because prices in the rainy season are low. However, farmers who have sufficient water and irrigation technology have the opportunity to irrigate their khat during drought season (khat may also be exasperating water shortages). This situation enables them to sell their khat at higher prices, which can fluctuate wildly. During this season these farmers have better bargaining power mainly because there is greater demand for khat than supply. The problem with easily perishable khat is that once it is collected, it is dead and cannot be carried back home, providing power to middlemen who manipulate prices and supply chains.

However, not all socio-ecological changes in the district are wholly negative. Changes in livelihoods over the last two decades that were discussed by community members in our research as (for the most part) “positive changes” included: a total shift from living in thatched houses to modern houses covered with corrugated sheets; increase in access to and use of agricultural technologies such as improved seeds, pesticides, fertilizers and water drawing motors; increase in television access to local and international news; increase in the number of rural residents who own small or larger vehicles, flour mills, mobile phones, and urban-based business activities; increase in use of high quality household facilities such as good mattresses, pillows, and electricity; increase in the number of small shops in rural villages; increase in access to modern health centres and roads; increase in construction of hand dug wells and use of pump-supported irrigation; and the gradual increase in the number of households that use latrines to dispose of human feces.

That said, life remains precarious for many people in Haramaya, especially for the majority of rural residents and villagers who have not necessarily benefited from these advances and, clearly from our many interviews, view progress with some skepticism, and would rather emphasize the difficult predicaments they find themselves in as they struggle for life.

### Ethical approvals

This study was funded by the Bill and Melinda Gates Foundation as a complementary grant to the USAID-funded “Feed the Future Innovation Lab for Livestock Systems” implemented by the University of Florida’s Institute of Food and Agricultural Sciences in collaboration with the International Livestock Research Institute. We received an IRB exemption from both the University of Florida and Haramaya University to conduct the work presented here.

## Results

Our results are presented in four sections. The first section (1) will discuss the basic socio-economic structure of village chicken production and reproduction in Haramaya, while the latter three sections will discuss different regimes of care for (2) chickens, (3) children, and (4) environments.

### Village chickens and reproductive politics

#### Poultry economics

Poultry are interwoven into the rural landscape and peasant economy of Haramaya district, albeit as a marginal economic practice to the dominant logic of khat production discussed above. As with other studies [26], poultry care is primarily a female space, part of gendered care routines, where women and girls actively care for chickens in many of the same ways they care for other household members. As one man succinctly summarized, *“Chickens listen to the sound of the wife, not the husband.”* While women do use chickens as a source of food, they are reared chiefly for income. Chickens provide independent income for women to negotiate household necessities, especially when they encounter health and financial problems.

Most households we spoke to currently or recently had chickens. Our household survey (*n* = 128) found that herd size was an average of six birds (standard deviation 3.4), with a maximum of 30 and participants self-reported an average of 12 eggs per week being produced per household (57% reported receiving 10 to 20 eggs per week).^1^ We also encountered many households who did not have chickens – most of these households had recently (within the last year) lost their flocks. Our observations also suggest that chickens are more often kept in lower socio-economic status households.

Chickens produce three primary economic benefits for people at village level: through eggs, chicken meat, and fertilizer. Due to scarce and limited resources, poultry are rarely eaten outside special events – egg production is by far their main economic output. Eggs are also seldom sold within rural villages or between households but instead are sold by women at local markets (5–10 km away). Buying eggs, and exchanging cash for them, is an activity that moves along rural-to-urban peasant market chains.

During our ethnographic research, people regularly laughed at the idea of buying eggs themselves and the idea of eating them. A common refrain was: “*We can’t afford to eat them. We sell them and buy other things.”* Eggs normally sell for between 4 to 5 Birr per egg (0.15–0.22$ USD), meaning that the average household could generate 50 to 100 Birr per week (1.80–3.60$ USD) from selling eggs. This is a not-insignificant amount of money; for example, farmers sell a kilo of potato at 3 Ethiopian Birr and poultry are much less labor intensive than khat or vegetable cultivation.

Chicken production still requires work, however. Ensuring that chickens produce a regular supply of eggs requires that poor, rural women invest time and effort into caring for them, alongside the care they already provide for their family, other animals, agricultural fields, and petty trades and businesses.

#### Reproductive politics

Part of this caretaker role involves governing the reproductive politics of hens: the sexual advances of, and competition between, roosters; the impulse of hens to want to hatch eggs and tend to chicks; and the choices about breed and genetic stock. If a woman was too “greedy” and took many eggs at the wrong time, hens were known to “rebel” and cease producing eggs in protest. If a healthy balance between producing eggs and offspring was not maintained, hens could become infertile, refuse sexual advances from cocks and even become “suicidal.” For example, one local (and relatively common and well-known) treatment for hens that are refusing to lay eggs, because they are “rebelling”, is to tie them upside-down for a day or two, to “discipline them.”

Hence, there are periods when hens do not lay eggs at all, as they care for their young or refuse to cooperate with their human masters.

Reproductive politics also extends to genetics and breed choice. In connection to this, studies have shown that the egg production potential of exotic hens like White Leghorns are more than five times those of local indigenous chickens [27]. But to benefit from this, birds need to be cared for “like a special thing”, which few families are able and willing to do. There are sensible reasons for this.

Improved breeds (like Sasso and Bovan Brown), and some exotic and crossbreeds, are referred to locally as the *Jazba* breed (the “confused breed”) because they are unable to protect themselves against predators like foxes, dogs, and owls. Instead of running away, they are perceived to *“allow themselves to be eaten.”* They are also known for their voracious appetite, more willing to scratch in farmlands and destroy people’s crops. They typically have problems hatching eggs and reproducing their flock, and are viewed as temperamental and fickle. As a consequence, women often are required to find indigenous breeds to hatch eggs of these birds, but this does not always work. Lastly, eggs of the improved breeds are up to two times as big in size but not as sweet as that of the indigenous breeds (also discussed also by [28]); household members mentioned that this can be improved by crossbreeding (with White Leghorns or Fayoumi).

**Fig. 2:**
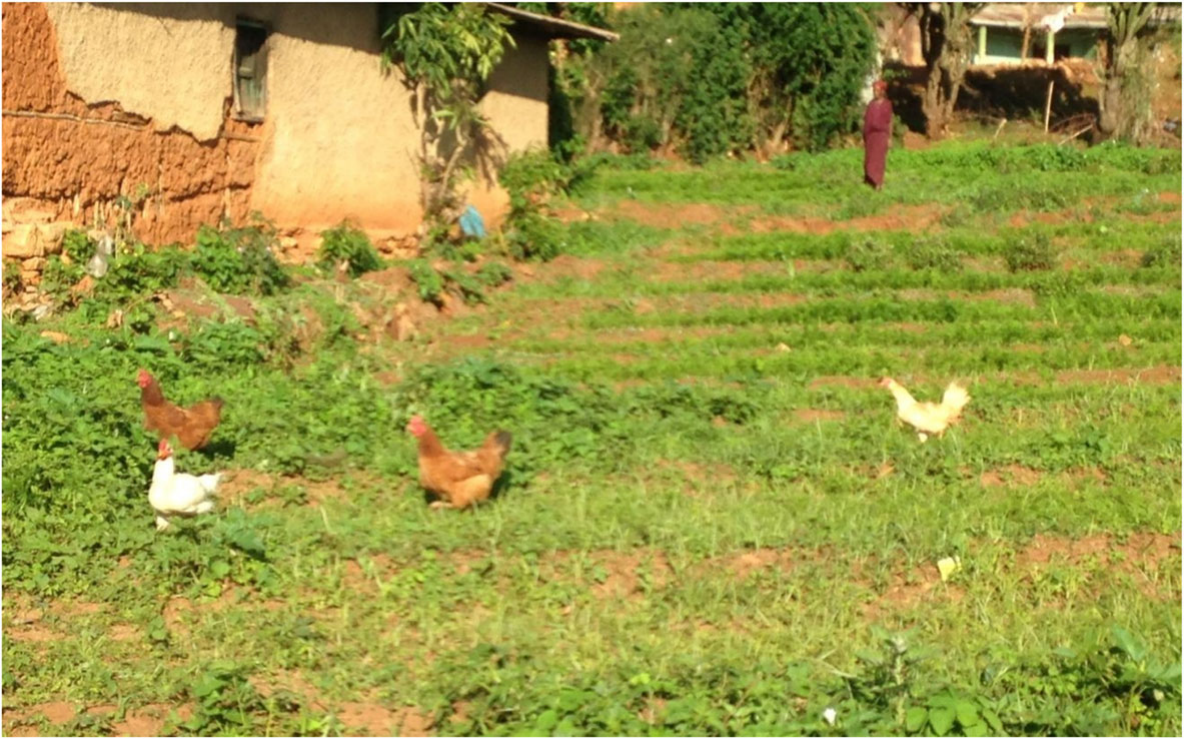
Indigenous chickens running through a field in Haramaya district

For these reasons, the majority of households keep local indigenous breeds. These birds follow what is called a *su’aallachuu* lifestyle (an “independent search for life”), scavenging in yards and bushy shrubs, where they require less care, attention, and resources to manage under prevailing conditions (Fig. 2).

### Caring for chickens: the social determinants of poultry health

Ensuring that chickens produce eggs, however, does require particular human-chicken regimes of care: chickens need some form of housing, in particular at night to protect them from predators, and they need to be monitored, for example released during morning and gathered back at dusk; they need to be supplied water and supplementary grain; they need attention when they are sick; their feces need to be cleaned; and they need to be managed to reduce herd conflict and, as noted above, to ensure their reproductive practices. Three particular care regimes stood out in our research as most important to egg production: feed, housing, and healthcare. The degree to which households deliberate about these regimes – intentionally, passively, or partially– has an important influence on egg productivity.

#### Chicken feed

Although chickens are largely free-ranging and obtain their feed through foraging, in some cases they are also nourished through supplemental feed that helps produce more and better eggs and offspring. Routines vary, and are often not consistent depending on the particular household. Wandering inside houses, visiting sleeping quarters and kitchens, women respond to the “pressure” of chickens’ search for sustenance and will provide them with an assortment of maize, sorghum, wheat, kitchen wastes (potato and carrot skins) and leftover food. Ideally this occurs once a day but there are seasonal influences and much variation.

During the rainy season, and to prevent crop damage around harvest time, chickens are made to stay closer to the homestead, and move around less. The “fear” of being eaten by predators (since tall crops can easily hide them) and the wet and cold weather also contribute to this reduced mobility. Women spoke about keeping chickens during the rainy season as a *mashaqqaaa* or *dhibkaaguddaa* (a “troublesome and big burden”) because of the difficulties in supplying feed. To make matters more challenging, the harvest period corresponds with a shortage of grain at household level (the preferred poultry feed), something that has become exasperated by the agricultural transition to khat, which has reduced the availability of grain and vegetable production. Most people believe the chicken population has decreased over the last 10–15 years in Haramaya due to this.

#### Housing chickens

**Fig. 3:**
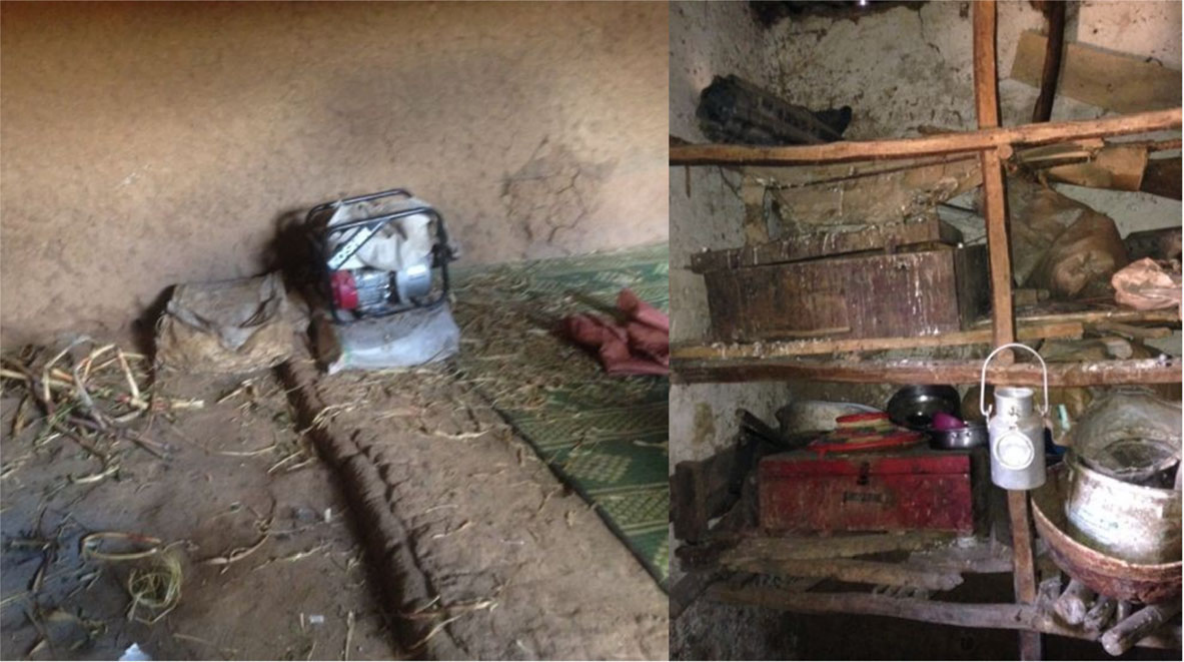
A typical division between human and animal sleeping quarters (left) as well as a chicken sleeping roost directly above the kitchen (right), note the high volume of chicken feces

The second major caring activity is housing. The Hararghe Oromo traditionally keep livestock inside at night but not typically in separate housing or cages (Table 1**).** Chickens often share the same room with sheep, goats, cows, calves, and donkeys, but also humans. In many cases, a small half-wall separates the animals from mats, carpets, and the occasional mattress where most families sleep together in one large room (see Fig. 3) Conditions can be rather unhygienic, but shared housing is influenced by cultural norms and expectations, a lack of building material, and the need to protect livestock from thieves and wild animals. Chickens perch on roosting bars, old containers, and pots, pans, and bags; laying hens and chicks sit in small boxes and baskets, concentrated in the family kitchen or storage room. They move in and out of sleeping areas and have access to human objects including kitchen utensils. They form emotional bonds with the women and children who care for them. Such close contact facilitates the spread of mites and, to a lesser degree, ticks and louses, and such bites can expose people to serious bacterial infections. To prevent this, a common practice (we estimate about 20% of households do this) used to reduce chicken-mite-human contact is for households to keep chickens in sacks at night. This also helps to collect chicken feces (for fertilizer) and ensures that roosters, once the dawn arrives, do not wake-up the family with their annoying cries.

**Table 1:** Where do chickens stay overnight?

	Number	Percent
In a separate place	18	141%
Together with humans	21	164%
Together with other animals	53	414%
Together with humans and other animals	36	281%
**Total**	***N*** **= 128**	**100%**

Physical boundaries, like fences, are very uncommon in these villages; chickens move freely between households. Conflicts only occur when they destroy seedlings and peck at vegetables, provoking social confrontation between neighbors and occasional flock poisonings. Although people restrict movement of cows and goats, only a minority keep poultry houses and cages, mainly those designed by extension programs from nearby Haramaya University. Restricting poultry movement is viewed as economically unfeasible due, primarily, to the costs of feed. But caging also has subtle negative connotations as a “chicken prison”, something that was also found by Harvey et al. [29] in Peru where free-roaming chickens were believed to be “healthier, happier and produce better meat and eggs.”

#### Poultry diseases

Of the poultry cages we saw, very few were still functioning. The most cited reason was that they had been “emptied by poultry epidemics” – know as *Dhukkuba lukkuu*. Many people we interviewed claimed that their current flocks were less than 2 years old because of this. Epidemics sweep into these villages every few years, demotivating households from building larger flocks. From our discussions, it was difficult to know what diseases these were exactly, although Newcastle disease is likely one of them (see [30]). With almost no veterinary services or access to poultry vaccines, sick birds are treated with onion and garlic, treatments that are not considered to be efficacious during outbreaks. If a few poultry in a flock become sick, or if there are rumors of an epidemic in neighboring villages, women sell their poultry to avoid losses (often keeping only a hen and her eggs, which are isolated from the group). This practice likely spreads the epidemic to neighboring areas.

### Caring for children: eggs, poverty and “dietary consciousness”

For women in Haramaya, there are many associations between managing children and managing chickens. These run along various care routines – from feeding, housing, teaching, supervising, and safeguarding. As with chicken production, narratives of scarcity dominated discussions about child nutrition and mediated how families view, seek, and use the most accessible protein source available: chicken eggs.

#### Malnutrition and eggs

Malnutrition is high in rural areas of the district and egg consumption is low. Over half (53%, *n* = 68) of households we visited in our survey had a child with notable symptoms of malnutrition. The prevalence of malnutrition was not different between villages. About 90% of respondents reported that their infants (under 2-year olds) had never consumed eggs. Of children younger than 10-years old, only 10% had consumed them in the previous week. The local adage was: *“I have never tasted eggs.”* However, at the same time, eggs and milk were understood to be essential priority foods for children and also for pregnant women. They were seen to make children “shine”, since they were *“body builders … they give strength, vigor and disease tolerance,”* as many women would say to us. Nutrition was also acknowledged as a mediator of personality: better-nourished children were observed to be physically, mentally, and emotionally more dexterous, joyful, and resilient (according to our many interviews). So what are the reasons for this disparity between nutritional knowledge and practice?

#### Parental fatalism

A dominant explanation involved a “fatalistic” perspective of many parents about how child growth occurs and about the role of the family in supporting this. In this view, once children have stood-up straight and can walk, any further cognitive and physical development is not dependent on parental intervention. A child has been endowed (*rizq*) with a certain personality and physical characteristics, and there is little that can be done to prevent abnormalities or shape psychosocial growth. According to this perspective, worrying too much about food or drink is seen as sinful, as an affront against God’s affairs. This perspective tended to be reinforced within strongly paternalistic families, ones with a much lower appreciation of the role of protein and eggs in child growth.

#### Poverty and malnutrition

A second, more nuanced, perspective highlighted the relationships between poverty, economic forces, and such nutritional fatalism. Here, the ability to care for children – and provide them with eggs – was mediated by noxious living conditions. A well-trodden local maxim is that “*the roots of malnutrition are poverty.”* Generally, animal-sourced foods are sold to generate income; only a small minority of households use them on a regular basis with the explicit goal of improving child health. We observed many malnourished children alongside skinny, malnourished mothers whose physical conditions revealed the intensity of their food shortages. We also encountered numerous instances of children who suffered from repeated bouts of diarrhea and ill-health that their parents ascribed to a lack of nutritious food. Lastly, households with a large number of children (common in the area) struggle to feed them, while the ever-increasing population also intensifies competitions among siblings over scarce resources. From this perspective, people may be well aware of the adverse impacts of under-nutrition on the physical, emotional, and behavior pattern of children, but they have little power, or capacity, to change it. In fact, malnutrition was viewed by many as increasing in Haramaya due to khat production driving a decrease in grain and vegetable production and to climate change drying up, literally, access to water.^2^

#### Social norms, food and living conditions

The third perspective, building on the others, stressed the ways in which social norms, interacting with living conditions, mediate child diets. For example, breastfeeding is a normative practice in the area, for a minimum of 6 months and up to 2 years old. But in practice, mothers are often prevented from fulfilling this: if a woman becomes pregnant she will typically stop breastfeeding (believing that this milk is no longer “good” for the child and that the fetus’ growth cannot be sustained while breastfeeding). Likewise, they will stop if they are busy in the field and in the market, without adequate social support. The rapid increase in khat production and women’s role at markets may exacerbate this separation between women and infants. And if women themselves are undernourished, milk production will stop naturally. Once a child is no longer exclusively breastfed, or when they can stand-up, they eat the same foods, at the same time, as those given to other family members. The diet of children under 1 year old, therefore, is largely made up of *laaffisoo* (a combination of *enjera* and local stew prepared from flax, potato, and carrots) with little protein or fruits. Other food include: potatoes, carrots, cabbages, bread, porridge, rice, pasta, beans, peas, maize, and pumpkins, as well as packaged foods like biscuits, mango juice, and artificial sweetener powder. There is a gendered routine to feeding that is also important here: traditionally women (and girls) eat after their husbands and male children are satisfied. A household might also not want to come across as “too urban” or “too showy” by practicing deliberate feeding of their infants and children, of *“fussing too much”* about it. And if they have many young children this may present its own problem about who gets access to eggs.

#### “Dietary consciousness”

A final perspective recognized the commonalities between the three other dominant views and stressed the ways in which they interacted, specifically in terms of how economic conditions and social structures produced and reinforced a “poverty of thinking.” In this view, even households with high quality khat fields and higher incomes were still selling their eggs rather than feeding them routinely to their children. This entails the importance of nutritional awareness, prioritization, and planning for improving dietary practices and leading healthy lifestyle. Some community members were able to verbalize this tendency as a proclivity to sell away good things such as egg, milk, and non-durable vegetables to purchase bad non-food items, such as adulterated oils, salt, gas, cigarettes, soda, and candies. In this view, knowledge about the value of eggs was increasing, spread particularly by health professionals during antenatal visits and child health visits, which was (slowly) driving changes in dominant nutritional attitudes. The empowerment of women was an important node in this: those who are more mobile and visit towns, have a smaller family size and are connected to modern media, would be more able and willing to re-orientate the dietary intake of their kids and to influence their peers, families, and networks.

In this last sense, people spoke about the need for “dietary consciousness” to spread in Haramaya: explicit food planning to ensure that children are provided with protein and diverse diets to address chronic child malnutrition, even in the face of difficult circumstances. Proper feeding is an important social value, and women who do not bother about the feeding habitats of their child are known locally as *islahe* (reckless and careless). But egg consumption has not yet become part of this “normal” diet, for many people.

### Caring for domestic environments: excrement, enteric bacteria and children

The care of children and chickens takes place, and is entangled within, a physical landscape inhabited by enteric bacteria and other microorganisms. Although we found that community perspectives strongly link poor hygiene, water, and sanitation to the ill-health of children, these risk perceptions do not extend to the contact between children and chicken feces.

#### Excrement and domestic spaces

**Fig. 4:**
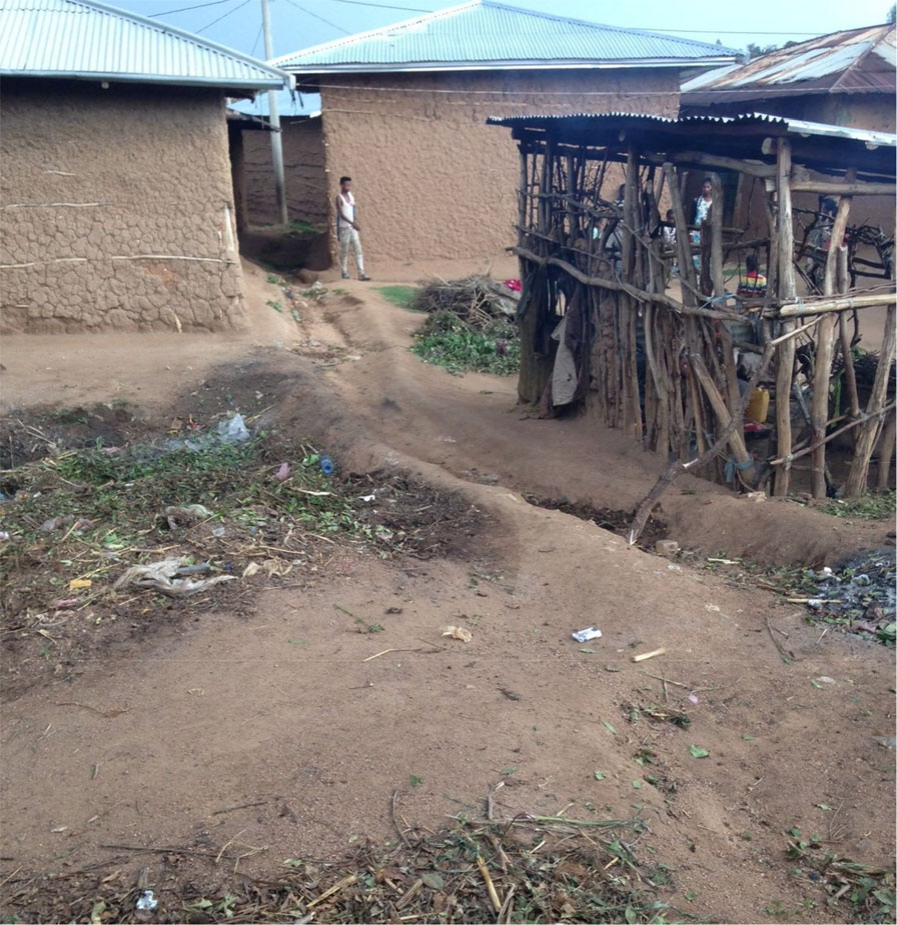
An example of a well-kept domestic environment, with garbage and feces swept into discrete dump piles; a few minutes after this picture was taken, a large group of young children began to play a game on the rubbish pile.

We found high levels of human and animal excrement around nearly every homestead we visited in Haramaya. During our household survey, for example, we systematically recorded this for 128 households: 73% had significant amounts (medium to high) of cattle, goat and sheep feces around the home environment, 55% had chicken feces and 31% human feces. We seldom encountered households without any fecal contamination (Fig. 4)**.**

Human feces were present typically behind the homestead and in bushy areas nearby. It is common that children defecate in the open, even in households with latrines, and many adults also defecate in the open when they are working on their farms, looking after animals, travelling, or when they do not have a latrine. Roughly half of the households we visited in the household survey had a latrine, and half did not. This fits with our general observations. The most common type (51%) was a traditional latrine made of mud, wood, and an iron sheet (see Table 2). However, most were poorly constructed: shallow, wobbly, and lacking a fixed concrete slab, walls, or doors, protected with torn sacks and plastic sheets. Community members stressed the fact that because households are often clustered together, it can be difficult to secure land at an appropriate distance from sleeping quarters to build a latrine. Lack of knowledge about construction techniques and access to materials is also a barrier. In response, people share latrines. Despite a strong cultural tradition of body washing and bathing (including before daily prayers), none of the observed latrines had a place for washing hands, and perceptions of the importance of hand-washing were generally low.

**Table 2:** Types of toilets used by households

Types of toilets	Frequency of HHs	Percent of HHs
Traditional latrine made of mud, wood, and iron sheet	34	27%
Tent covered latrine	8	6%
Ventilated improved pit latrine	3	2%
Plastic covered latrine	11	9%
Grass covered latrine	5	4%
Uncovered latrine	3	2%
No latrine	64	50%
Total	*N* = 128	100%

#### Perceptions of human and animal feces

Psychosocial responses to feces differentiated animal excrement, considered tolerable waste, from human feces, seen as intolerable waste. This had implications for emotions of disgust, ideas of impurity, and prevention practices. Human feces, and also to some degree urine, were seen as unsafe and impure. A person that came into contact with them should not perform Islamic prayers, for example, until they were ritually cleaned. Human feces are known to cause health problems, especially diarrhea, worms, vomiting, and poor appetite, if it enters water sources or food through hand-to-mouth contact. For these reasons, households will frequently remove child feces much more regularly than animal feces, spread by dogs, cats and domestic livestock who roam freely between households.

By contrast, our fieldwork showed that people in Haramaya do not generally link exposure to animal feces to human health problems. In fact, community members have little awareness of the risks of zoonotic infections or health hazards associated with human-animal co-existence in general. Low-risk perceptions of animal feces were partially due to their economic and ecological benefits; feces from cows, donkeys, and chickens are particularly valuable in increasing soil fertility. Cow dung is used for cooking, building, and for manure, whereas chicken feces is used mostly to enrich khat production and is considered the most highly-valued manure. There is a gendered division of labor with exposure to chicken feces: women and girls manage the wet feces, by daily sweeping, cleaning and collecting, whereas men are responsible for moving the dry feces, from compost piles or sacks next to the homestead, to agricultural fields. Compost piles and garbage pits are only a few meters away from the kitchens and houses where people sleep, eat and play. And dumping grounds are sites where flies bridge the world of feces and food.

Chicken feces fall between the categories of tolerable and intolerable waste. They are seen as more nocuous than other animals because of the scavenging behavior of poultry – they are “dirty” and “disgusting” creatures that have a “dirty diet”: they eat human feces, animal dung, and worms. Because of these associations, people report that the smell of chicken feces is repugnant. This is also the reason why most people in our study villages do not eat the gastrointestinal contents of chickens (but do for other animals) and will wash chicken carcasses according to a specific hygienic routine; some even report washing chicken eggs for this reason. We did not encounter anyone who was able to name any particular diseases caused by chickens or animal feces in general, or any personal experiences, or stories from such infections. However this association has generated a vague and widely held idea that chicken feces are dangerous and can cause health problems. This is thought to occur when chickens touch kitchen utensils and steal food from plates and other sources.

#### Child and chicken feces

It is children, of course, who are most at risk from the enteric pathogens spread by chicken feces that contaminate the soil and surfaces around the homestead. Contact between children and chickens can be intimate: children chase and catch chickens; sometimes chickens try to eat from their dinner plates; some kids sleep next to chickens; and some help prepare and clean boxes where hens lay their eggs. There is also a tendency for some children to consume raw eggs, both due to taste and as a treatment for dry cough.

But it is mostly through environmental exposures that enteric pathogens are transmitted. Without safe playing spaces, and often carried on the backs of their older female siblings, children are constantly on the move – on hands, knees, and barefoot. As with Ngure et al.’s [17] study in Zimbabwe, childhood mouthing and exploratory play placed children at high risk of fecal contamination. We observed children, on numerous occasions, putting the following objects in their mouths: their fingers, food off the ground, khat leaves, insects, animal and human feces, metal objects, bird feathers, house utensils, plastics, and cardboard. Such objects move freely between the ground and the mouth. Women emphasized that their vigilance to monitor these microbial encounters was often low, due to their engagement with domestic chores and multiple other responsibilities, although we observed that some are more observant than others and will wipe the object or hand if they see it, with water or a cloth – but not with soap.

## Discussion and conclusions

In this exploratory study, we investigated the ways in which village-level chicken production, child diet, and environmental hygiene conditions intersect and interact as they produce health and illness in Haramaya District, Ethiopia. In this final section, we reflect on our findings and their relevance for agriculture, health, and poverty alleviation in the region.

Emerging evidences show that animal feces, especially from chickens, transmit a host of dangerous enteric pathogens such as *Campylobacter spp.* and nontyphoidal *Salmonella enterica* [15] and, while the science is still evolving, that these microbial exchanges are responsible for some proportion of global childhood stunting [20, 31]. In this study, we found that local explanatory models of disease did not associate animal feces with human illness despite widespread fecal contamination. This is in line with other studies, where perceptions of zoonotic disease risk often center on handling or consuming uncooked chicken meat and sick or dead animals, and not animal feces [29, 32]. In contrast, human feces are widely known as sources of disease, motivating households to use latrines or at least defecate away from the immediate homestead area. The exception is young children, whose feces intermix with those of animals around domestic landscapes.

The utilitarian value of livestock dung, including the use of chicken manure on khat fields, served to dampen or supersede the vague connections that existed, for many, between chicken feces and mites, poultry scavenging behavior and human emotions of repulsion and risk. Perceptions of chicken feces oscillated between tolerable and intolerable waste, and between risk (for health) and benefit (for manure). Daily sweeping of feces and the collection of garbage into compost piles served to reduce exposure, but challenges of daily life ensured the partiality of these efforts and high exposure of young infants and children as they played in the dirt.

If animal feces are bad and good – pathogenic and salubrious – how should risk be conceptualized and communicated? Clearly, education efforts focused on the dangers of chicken feces in Haramaya district would find intuitive support, due to existing perceptions and experiences that already associate it with psychosocial sentiments of dirt, disease, and danger. Risk communication scholars oscillate between carrots and sticks, with proponents of fear-based communication confident in the efficacy of their strategies and opponents, focused on more encouraging strategies such as positive deviance, equally confident that a reliance on fear alone can backfire [33]. During the West Africa Ebola epidemic, for example, fear-based messaging on the dangers of hunting and consuming bushmeat was met with resistance because they did not fit within local experiences of disease or socioeconomic pressures and livelihood realities [34]. Like chickens, bushmeat has a double role as both a threat to health but also as a source of protein to nutrient-deficient populations. Focusing too much on the health risks (child stunting), while overlooking positive livelihood dimensions (childhood egg consumption and manure) may not be the most effective communication approach, and could even stigmatize chicken keeping in some communities if pursuit with too much fervor.

In this paper, we have used the concept of *regimes of care* to illustrate the connections and disconnections between the health of chickens, children, and domestic environments. Our purpose has been, on the one hand, to stress how people in Haramaya viewed the caring of children and chickens in somewhat synonymous or at least comparable terms. Caring for both is the domain of women and girls, who assume caretaker roles in a traditionalist Oromo society. Women have the duel task of ensuring that the beneficial interactions between children and chickens are maintained, notably in egg production and consumption, while the negative consequences of child exposure to environmental risks, like chicken feces, are reduced. In this sense, *regimes of care* refer to deliberate, conscious acts, or a set of practices, that govern the social determinants of children and chicken health. A focus on *regimes of care* for zoonotic disease management helps us locate spaces of interaction beyond a focus on negative risk and towards appreciating how health and disease are joined together in acts of interspecies caring and neglect.

As we have shown, *regimes of care* are made-up of innumerable daily chores, tasks, and decisions, operating in a broader biosocial context. We described that continuous egg production is a fragile activity, with its own *reproductive politics* as women manage the needs of hens: in the sexual advances of cocks, the demands of hatching chicks and in the choice of breeds. The quality of eggs, however, were largely a result of breed but also the ability for women to negotiate scarcity as they sought to manage what we called the “social determinants of poultry health”: supplemental feed, appropriate housing, and healthcare. Hence, scarcity maintains chicken production as a fragile and inherently “risky” activity.

In this sense, appreciating care regimes is also a useful approach to understanding barriers to change. With the emergence of avian influenza in Southeast Asia, poultry biosecurity interventions have become a major focus of global animal health but national policy guidelines and programs have remained surprisingly disconnected to the contexts of small-scale rural farmers [35].

This study has added some texture to the challenges of implementing poultry projects in contexts of scarcity, given the political economy of peasant livelihoods in one district of rural Ethiopia. With the reduction in cereal production due to a dominant khat-based economy, and increasingly erratic climatic variation, access to feed will remain a major problem into the future. Access to veterinary care is also an important barrier, with frequent poultry epidemics serving to demotivate households in keeping larger flocks, something that could be addressed through low-cost village animal health worker networks, although the viability of this kind of market is uncertain [36]. Feed and veterinary care are both precursors to better poultry housing and improved genetic breeds, as well as greater segregation between bird feces and children.

It is also important to note that the evidence on the effectiveness of caging poultry on enteric diseases are mixed. Oberhelman et al. [37] reported the results of a poultry caging case-control intervention design in Peru on human and chicken prevalence for *Campylobacter*. Over a 17-month period, they found that rates of *Campylobacter*-related diarrhea in the intervention group were significantly greater than in the control group, suggesting that caging may actually enhance infection.^3^

The idea of *dietary consciousness*, which we introduced above, offers another way to think about intervening, focused on social empowerment and behavior and attitudinal change. This term was developed through our ethnographic data, as we heard the ways that local people spoke about the disconnections between carelessness for child diets and informed, deliberate, and proactive dietary regimes. *Dietary consciousness* presupposes a sense of cognitive internalization that child diet, especially in the first 2 years of life and with access to protein, determines a child’s future.

Improving *dietary consciousness*, through mother-centric education and social empowerment, is an urgent priority to improve childhood health in Haramaya district. As we described, malnutrition is high while chicken egg consumption is low, with an assortment of attitudinal, behavioral, economic, and social factors involved. Women and children are more exposed to wet chicken feces, further highlighting the need for a gendered approach. But is focusing education and social empowerment only on women the most effective strategy? Reflecting on the Zika epidemic in Peru, Guerra-Reyes and Iguiniz-Romero [39] questioned the exclusion of men in risk communication because sexual and reproductive health decision-making remained under their control. In Haramaya, men are considered the “pillar of the house”, and finding ways to raise their involvement in child and maternal health is important for all family members. In our study, young men also expressed great interest in increasing chicken production as a strategy to diversify incomes, in the context of a continued decrease in landholdings, ecological challenges to crop agriculture, and the volatility of the khat cash-crop market. Hence, some masculine involvement seems a necessary part of shifting decision-making, if women are to sustainably redirect the income they would otherwise obtain by selling chicken eggs towards improving the cognitive and physical potential of their human offspring.

Any intervention on childhood nutrition needs to address the culture of food and feeding as they relate to childhood dietary practices. It will require emphasizing the need for “special foods” for children (like eggs), including newborns, as well as tackling maternal under-nutrition. This requires novel communication strategies based on deliberate theories of change, such as the Behavior Centered Design approach proposed by Aunger and Curtis [40]. Finding ways to communicate the connections and disconnections between chickens, children, and domestic environments may be a novel approach, linking awareness of food insecurity, vulnerability to disease, nutritional deficiencies, and the future psychosocial development of the family and community.

### Study limitations

As with any study, our observational research had several potential limitations. The first involves the *representativeness of our data*. With our ethnographic approach, we had to balance depth of research (time in specific locations – 12 weeks in total) with the number of sites we selected (16 villages in 4 kebeles), which may have limited the level of rapport the field team had with community members. The household survey (*n* = 128) was not calibrated for statistical significance and would have benefited from a more robust larger-scale survey; however a follow-on cross-sectional questionnaire study conducted by our team (unpublished data) involved 102 children randomly selected in Haramaya largely triangulated and confirmed our findings. The second issue involves the *large number of topics* we covered. This was a challenge given the 12-week time period of the study. As an exploratory study, and the first in a number of planned epidemiological and intervention studies, this was an inevitable result of the broad range of issues involved in the CAGED project. We attempted to mitigate this by covering topics thematically (3 themes and 10 topics) in a systematic fashion but this was sometimes difficult. Most importantly, this limited our data on child health conditions and local conceptualizations of digestion and gut health; this important data was partial and incomplete and would have required more extensive ethnographic data collection and was excluded from this paper, despite its importance. The 12-weeks of fieldwork also limited our ability to understand trends over time on chicken and egg production (which would benefit from a longitudinal study) and to gain a more robust understanding of community change dynamics and their connection to political economy. The third issue involved the fact that our observational data was *not directly linked to epidemiological data*, which limits our ability to adequately evaluate the level of disease risk from chicken feces and the various contact interfaces we explored. At this point, the causative links between *Campylobacter* and child stunting remain hypothetical and uncertain. As of late 2019, the ongoing formative epidemiological research in Haramaya has not fully confirmed the primary hypothesis underlying the CAGED study: that exposure to *Campylobacter* bacteria from chickens is a major driver of EED and stunting. Speciation of the *Campylobacter* population in the feces of 11–15 months old children by shotgun metagenomic sequencing suggested that while chicken-associated species are common, there are equally prevalent species that are commonly associated with mammalian hosts, particularly ruminants. Further work is ongoing.

## Data Availability

N/A

## Abbreviations

CAGED: Campylobacter Genomics and Environmental Enteric Dysfunction
DALYs: Disability Adjusted Life Years
DHS: Demographic and Health Survey
EED: Environmental enteric dysfunction
LMP: Ethiopia’s Livestock Master Plan
RCT: Randomized control trial
WASH: Water, sanitation and hygiene

## Footnotes

1 There were seasonal variations: with more eggs available during the dry season due to feed availability and fertility patterns.

2 However, there is some counter-evidence to this perspective that indicates that khat sales may be associated with increased dietary diversity, but this relationship needs to be studied to better understand how that relationship is mediated by other dynamics (markets, seasonality, rains, socioeconomic status, etc.).

3 Caveats to these findings included the fact that cages were located near family living quarters, often on the roof or rear patio of the home, and hand washing was low among household members. This can also be understood by the dynamics of infection and immunity, where models suggest that disease occurrence can increase with the reducing force of infection [38].
